# Immunomodulatory Effects of Cinobufagin on Murine Lymphocytes and Macrophages

**DOI:** 10.1155/2015/835263

**Published:** 2015-11-19

**Authors:** Yang Yu, Hui Wang, Xianhua Meng, Lu Hao, Yue Fu, Linlin Fang, Dan Shen, Xiaomeng Yu, Jingshung Li

**Affiliations:** Liaoning Medical University, Jinzhou 121001, China

## Abstract

Cinobufagin (CBG), a major bioactive component of the traditional Chinese medicine ChanSu, has been reported to have potent pharmacological activity. In this study, we aimed to elucidate the effects of CBG on the activity of immune cells in mice. Peritoneal macrophages and splenocytes from mice were prepared and cultured in RPMI1640 supplemented with 10% fetal bovine serum. Concanavalin (ConA), lipopolysaccharide (LPS), and CBG (0.0125, 0.05, 0.15, or 0.25 *μ*g/mL) were added to the culture medium, and the phagocytic activity of macrophages was detected by MTT assays. Additionally, lymphocyte secretion of interleukin- (IL-)2 and IL-10 was detected by enzyme-linked immunosorbent assay, and the cell cycle distribution and cell surface markers were detected by flow cytometry. Our results demonstrated that CBG promoted lymphocyte proliferation; this effect was suppressed by combined treatment with ConA or LPS. Moreover, CBG also significantly improved the CD4^+^/CD8^+^ ratio in spleen lymphocytes and increased the percentage of spleen lymphocytes in the S phase. Finally, we found that CBG enhanced the secretion of IL-2 and IL-10 and increased the phagocytosis ability of macrophages. In summary, CBG could enhance activity of immune cells.

## 1. Introduction

Lymphocytes are an important part of the acquired immune system, and many lymphocytes are derived from the spleen, which is the body's largest immune organ, comprising one-quarter of the body's lymphatic tissue. Indeed, the spleen is the hub of cellular immunity and humoral immunity. T cells are primarily responsible for regulating cellular immunity, and splenic T cells account for 25% of the total number of T lymphocytes in the systemic circulation. These cells, which can regulate the distribution of peripheral blood T cell subsets, include helper T (Th) cells and cytotoxic T (Tc) cells [1]. T cells are able to release proinflammatory cytokines, which are responsible for the recruitment of effector cells, including macrophages, neutrophils, and eosinophils. There are several subsets of Th cells, the most common of which are Th1 and Th2 cells; these subtypes are defined based on differences in function and secretion of cytokines after identifying antigen [2, 3].

In addition to T cells, other types of immune cells have important roles in the immune system. For example, almost all tissues contain macrophages, which play a critical role in the immune system, with functions in both innate and acquired immunity [4]. Phagocytic activity, in which cells engulf foreign pathogens and other cells, is the most important function of macrophages. The phagocytic capacity of macrophages is a direct reflection of immune function in infants.

ChanSu is a traditional Chinese medicine prepared from toad skin that has been used in China for a long time, with the earliest recorded use published in “Shen Nong's Herbal Classic.” ChanSu has been used extensively to treat a variety of diseases, and the chemical composition of ChanSu comprises steroidal cardiac glycosides, indole alkaloids, and derivatives [5]. Animal experiments and clinical data have shown that ChanSu has many pharmacological effects, including alteration of cardiac function, alleviation of cough and asthma, anti-inflammatory effects, antitumor effects, antiradiation effects, enhancement of immunity, and analgesic effects [6–8].

**Figure 1:**
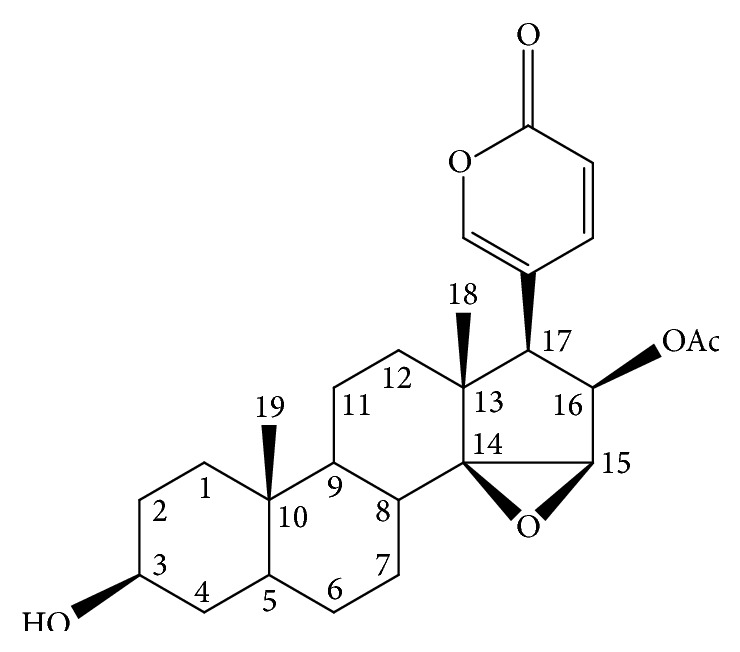
Structure of cinobufagin.

ChanSu is made from the skin of Bufo bufo gargarizans Cantor [9], and the main component of this extract is cinobufagin (CBG; Figure 1) [9], an important steroid. CBG has recently been shown to have antitumor activity, inducing apoptosis in prostate cancer cells [10] and inhibiting the migration and proliferation of human hepatic cells in vitro [11]. Moreover, several studies have examined the immunomodulatory effects of CBG in animals and have shown that CBG has protective effects against cyclophosphamide-induced damage to the spleen and thymus in mice. CBG also increases the T and B lymphocyte stimulation index, promotes phagocytosis by macrophages, and enhances metabolism in peritoneal macrophages. However, the specific effects of CBG on immune system function remain unclear.

Therefore, in this study, we examined the effects of CBG on immune cells and immune system function. Our data provide a theoretical basis for the clinical development and utilization of CBG.

## 2. Materials and Methods

### 2.1. Materials

BALB/C mice and* Staphylococcus aureus* (AB91093) were provided by Liaoning Medical University. CBG was purchased from Chengdu Mansite Biological Technology Co., Ltd. (Chengdu, China). RPMI1640 and fetal bovine serum (FBS) were purchased from Gibco (Beijing, China). Concanavalin (ConA), lipopolysaccharide (LPS), 3-(4,5-dimethylthiazol-2-yl)-2,5-diphenyltetrazolium bromide (MTT), and propidium iodide (PI) were purchased from Sigma (St. Louis, MO, USA).

### 2.2. Preparation of the Staphylococcus Suspension

*S. aureus* was activated and cultured at 37°C for 18–24 h. Single colonies were then picked and inoculated in fresh broth. Cells were further cultured at 37°C for 18–24 h and then diluted with broth to 10^7^–10^9^ CFU/mL.

### 2.3. Isolated Splenocytes

Mice were sacrificed by cervical dislocation and disinfected with 75% alcohol. Spleens were then removed and rinsed with RPMI1640 medium. Spleen tissue was homogenized in RPMI1640 and collected. Tris-NH_4_Cl was then used to remove the red blood cells, and cells were pelleted by centrifugation. The cells were then cultured in RPMI1640 medium containing 10% FBS in a 37°C incubator with 5% CO_2_. The medium was refreshed when a color change to yellow was apparent.

### 2.4. Preparation and Purification of Peritoneal Macrophages

Mice were sacrificed by cervical dislocation and disinfected with 75% alcohol. The abdominal cavity was then rinsed with RPMI1640 medium, and abdominal cavity washings were collected. Cells within these washings were precipitated by centrifugation and cultured in RPMI1640 medium containing 10% fetal calf serum in a humidified atmosphere at 37°C with 5% CO_2_. Trypan blue staining of viable cells revealed that 95% of cells were viable, and the cell density was adjusted to 5 × 10^6^ cells/mL. These cells were considered peritoneal macrophages. One hundred microliters of the peritoneal macrophage suspension was plated in each well of 96-well microplates. The cells were cultured in RPMI1640 medium containing 10% fetal calf serum in a humidified atmosphere at 37°C with 5% CO_2_ for 12 h. The cells were then washed with serum-free RPMI1640 medium.

### 2.5. Analysis of Lymphocyte Proliferation by MTT Assays

Cell suspensions (200 *μ*L, containing 1 × 10^5^ cells/well) were plated in each well of 96-well microplates. Cells in the logarithmic growth phase were treated with different concentrations of CBG or CBG and ConA in complete RPMI1640 medium. Control cells were left untreated. Four replicates were prepared for each treatment. After the incubation period, cells were treated with MTT (20 *μ*L/well; 5 mg/mL in phosphate-buffered saline [PBS]) and were then cultured for another 4 h. The supernatants were discarded, and 200 *μ*L DMSO was added to each well. The samples were incubated in the dark for 30 min and then swirled to mix. The Absorbance (*A*) at 570 nm was measured using an enzymatic plate reader. Experiments were repeated three times. The stimulation index (SI) was calculated as the *A* value of the experimental treatment divided by the *A* value of the control treatment.

### 2.6. Detection of Spleen Lymphocyte Subsets by Flow Cytometry

Cells were plated in 12-well cell culture plate at 1 × 10^6^ cells/well in complete RPMI1640 medium containing different concentrations of CBG and then cultured for 48 h. For flow cytometry analysis, cells were reacted with anti-CD4 and anti-CD8 antibodies for 1 h at 4°C in the dark and then subjected to flow cytometry analysis immediately.

### 2.7. Analysis of Cell Cycle Distribution by Flow Cytometry

Cells were collected by centrifugation at 1000 ×g for 10 min at 4°C. Cells were then washed with precooled PBS twice, resuspended in ice-cold 70% ethanol (v/v), and kept at 4°C overnight. After fixation, cells were pelleted, washed twice with PBS, stained with PI solution (0.05 mg/mL PI, 0.02 mg/mL RNase, 0.585 g/mL NaCl, 1 mg/mL sodium citrate, pH 7.2–7.6) at 4°C for 30 min, and then detected by flow cytometry immediately.

### 2.8. Enzyme-Linked Immunosorbent Assay (ELISA)

Cell suspensions (200 *μ*L containing 1 × 10^5^ cells) were plated in each well of 96-well microplates. Cells were treated with CBG and cultured for 48 h. Cytokine levels in the cell culture supernatants were then detected by ELISA.

### 2.9. Analysis of Macrophage Phagocytosis by MTT Assay

Cell suspensions (200 *μ*L containing 1 × 10^5^ cells) were plated in each well of 96-well microplates. In the logarithmic growth phase, cells were treated with CBG and then incubated with* S. aureus* in the presence of complete RPMI1640 medium. Untreated cells were used as controls, and four replicate wells were prepared for each treatment. After the incubation period, cells were treated with MTT (20 *μ*L/well; 5 mg/mL in PBS) in each well and then were cultured for another 4 h. The supernatants were discarded, and 200 *μ*L DMSO was added to each well. The samples were incubated in the dark for 30 min and then swirled to mix. The Absorbance (*A*) at 520 nm was measured using an enzymatic plate reader. Experiments were repeated three times.

### 2.10. Statistical Analysis

All experiments were repeated at least three times, and the results are expressed as means ± standard deviations (SDs). Data were analyzed using the GLM procedure in Statistical Analysis System software (SAS Inc., Cary, NC, USA) and compared with Duncan's multiple comparison test. Differences with *P* values of less than 0.05 were considered statistically significant.

## 3. Results

### 3.1. Effects of CBG on the Proliferation of Spleen Lymphocytes

**Figure 2:**
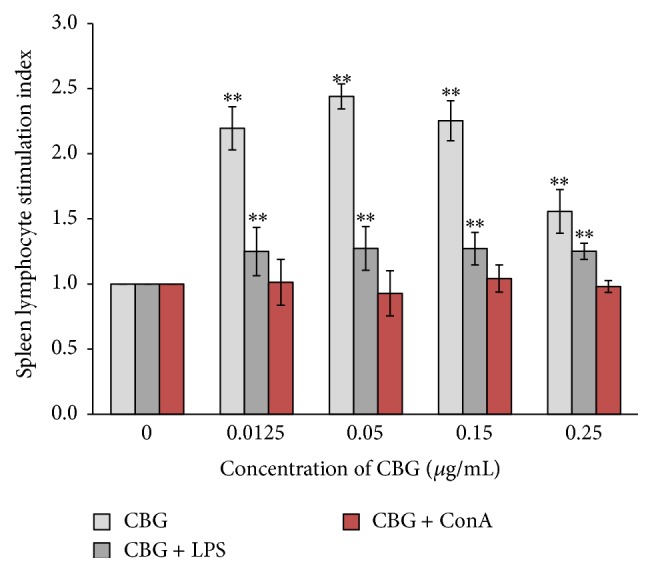
Effects of CBG and LPS on the proliferation of spleen lymphocytes. Statistical significance to corresponding control is marked with (*∗∗*) (*P* < 0.01).

First, we analyzed the effects of CBG on the proliferation of lymphocytes isolated from mouse spleens. CBG cause a significant, concentration-dependent increase in the stimulation index compared with that in the control group. Interestingly, as the concentration of CBG increased from 0.0125 to 0.05 *μ*g/mL, the stimulation index increased. In contrast, when the concentration of CBG increased from 0.05 to 0.25 *μ*g/mL, the stimulation index was decreased. These data suggested that CBG may have dual effects on the proliferation of spleen lymphocytes (Figure 2).

**Figure 3:**
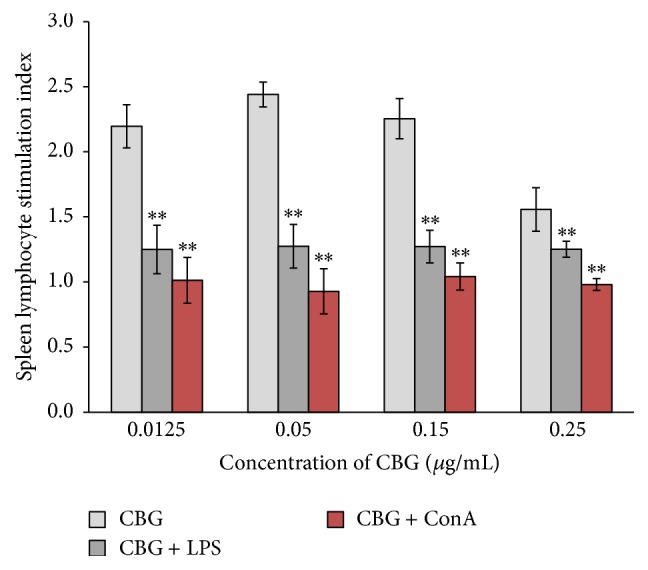
Effects of CBG and ConA on the proliferation of spleen lymphocytes. Statistical significance to corresponding control is marked with (*∗∗*) (*P* < 0.01).

Combined treatment with CBG and LPS caused a significant increase in the stimulation index compared with that of the control group. However, the stimulation index following the combined treatment was not as high as that following CBG treatment alone, suggesting that CBG and LPS did not have additive effects on the proliferation of spleen lymphocytes (Figure 2). In contrast, combined treatment with CBG and ConA did not result in a significant increase in the stimulation index (Figure 3).

### 3.2. Effects of CBG on the Expression of CD4 and CD8 in Spleen Lymphocytes

**Figure 4:**
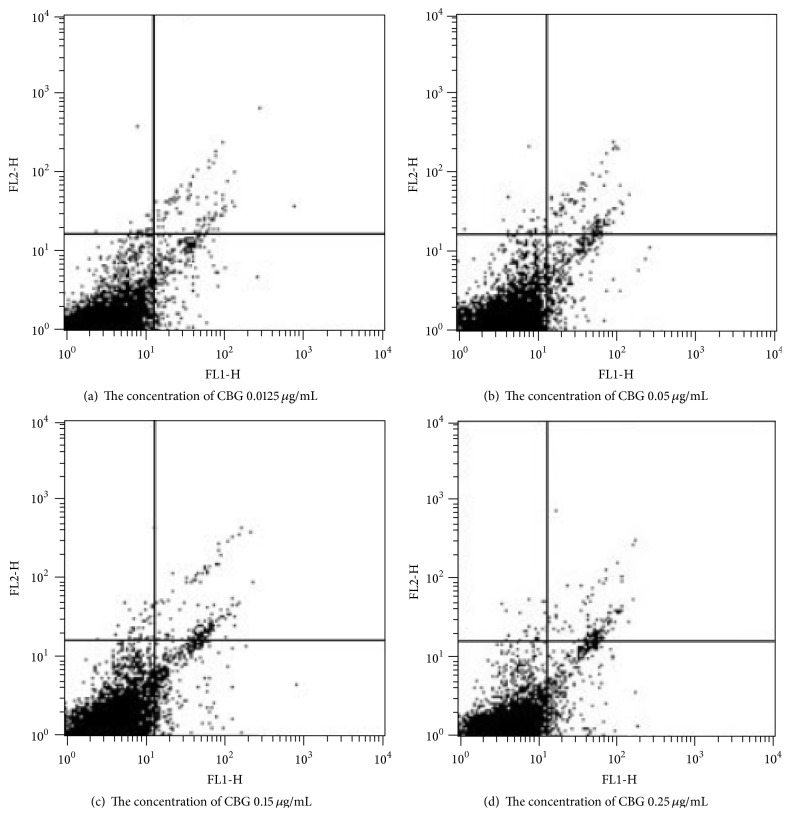
Effects of CBG on the expression of CD4 and CD8 in spleen lymphocytes.

Interestingly, treatment with CBG resulted in increased percentages of CD4^+^/CD8^−^, CD4^−^/CD8^+^, and CD4^+^/CD8^+^ spleen lymphocytes compared with those in the control group. The CD4^+^/CD8^+^ ratio can reflect the immune status; thus, our results showed that CBG may act as a positive regulator of immunity (Table 1 and Figure 4).

### 3.3. Effects of CBG on the Cell Cycle Distribution in Spleen Lymphocytes

**Figure 5:**
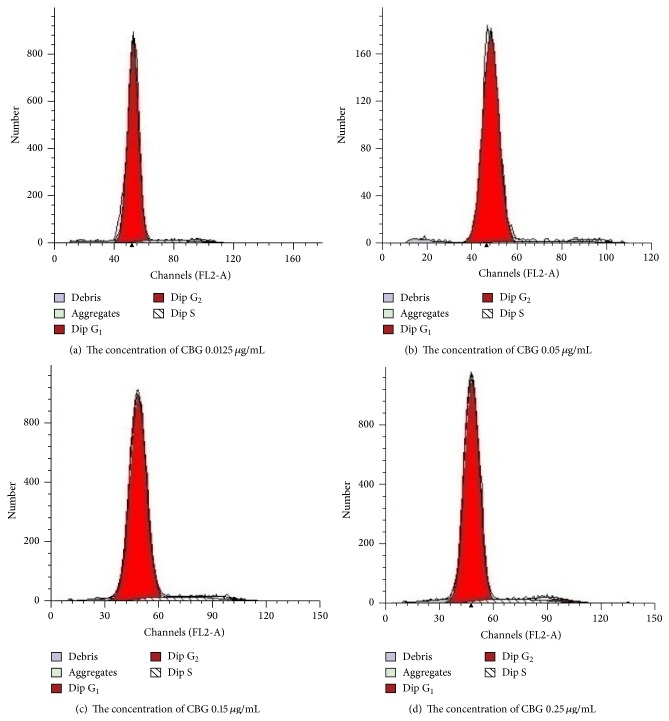
Effects of CBG on the percentage of lymphocytes in the S phase. Different letters indicate significant differences between treatments.

Next, we analyzed the effects of CBG on the cell cycle distribution in spleen lymphocytes. We found that CBG increased the percentages of spleen lymphocytes in the S phase of the cell cycle, suggesting that CBG could promote lymphocyte transformation from the G_0_/G_1_ phase to the S phase. These effects appeared to be dependent on the concentration of CBG; increased S-phase accumulation was observed as the concentration of CBG was increased. However, after reaching a certain concentration, the percentage of cells in the S phase decreased as the concentration of CBG continued to increase, suggesting that CBG may mediate cell proliferation by affecting the cell cycle distribution, particularly accumulation in the S phase (Table 2 and Figure 5).

### 3.4. Effects of CBG on Cytokine Secretion from Spleen Lymphocytes

**Figure 6:**
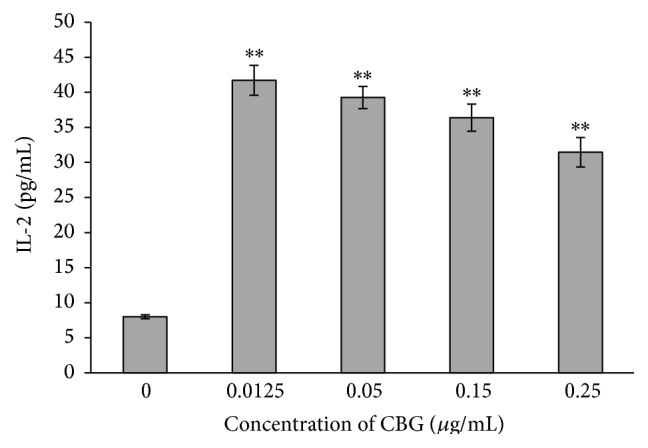
Effects of CBG on IL-2 secretion from spleen lymphocytes. Statistical significance to corresponding control is marked with (*∗∗*) (*P* < 0.01).

**Figure 7:**
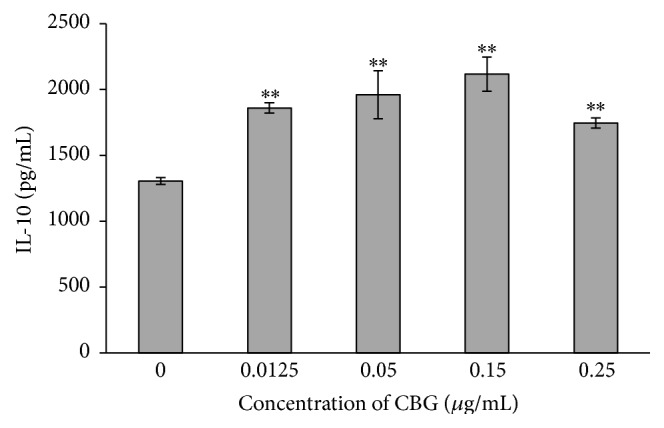
Effects of CBG on IL-10 secretion from spleen lymphocytes. Statistical significance to corresponding control is marked with (*∗∗*) (*P* < 0.01).

To further examine the mechanisms through which CBG affects immune system function, we examined the effects of CBG on cytokine secretion. CBG enhanced the secretion of IL-2 and IL-10, representative Th1 and Th2 cytokines, in a concentration-dependent manner. Secretion of IL-2 decreased as the concentration of CBG increased, while secretion of IL-10 initially increased as the concentration of CBG increased and then decreased as the concentration of CBG rose above a certain point. These data again supported the dual effects of CBG on IL-10 secretion from lymphocytes (Figures 6 and 7).

### 3.5. Effects of CBG on Phagocytosis by Macrophages

**Figure 8:**
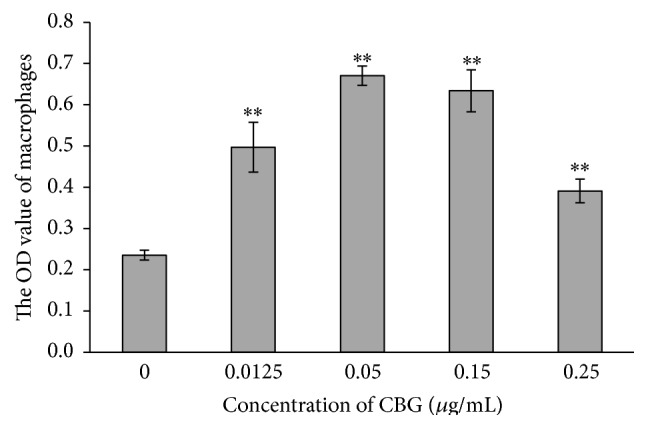
Effects of CBG on the phagocytic activity of macrophages. Statistical significance to corresponding control is marked with (*∗∗*) (*P* < 0.01).

**Figure 9:**
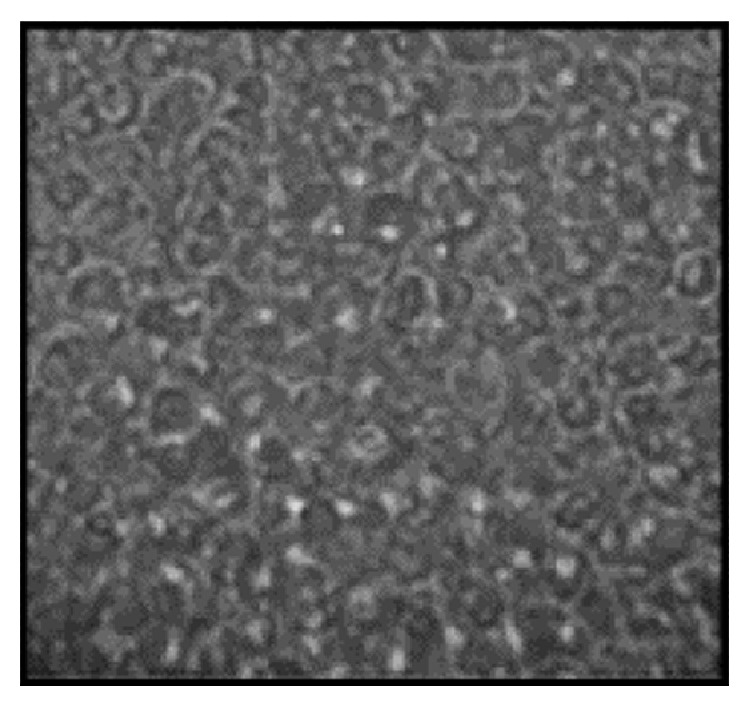
A macrophage swallowing* Staphylococcus aureus*.

Finally, we assessed the functional role of CBG in the immune system by examining the effects of CBG on phagocytosis by macrophages. Our data demonstrated that CBG significantly enhanced the phagocytic activity of macrophages (Figures 8 and 9).

## 4. Discussion

In this study, we aimed to examine the effects of CBG on immune system function in lymphocytes isolated from the spleens of mice. Our data demonstrated that CBG exerted substantial effects on immune cell functions in a concentration-dependent manner. These data provide important insights into the mechanisms through which CBG functions as a clinical therapeutic.

Th1 and Th2 cell subsets secrete different types of cytokines, resulting in the functional differences between the cell types. Th1 cells secrete IL-2, interferon (IFN)-*γ*, and TNF-*α*, which primarily mediate the immune response. In contrast, Th2 cells secrete IL-4, IL-6, and IL-10, which primarily mediate the humoral immune response. In order to maintain normal cellular and humoral immunity, Th1 and Th2 cells function to maintain a state of dynamic equilibrium [12]. B cells are primarily responsible for regulating the humoral immune response, and splenic B cells account for about 55% of the total number of lymphocytes. B cells also secrete immunoglobulin Ig under antigen stimulation [13].

Macrophages have antigen-presentation functions, working to process and present antigens to immune effector cells, thereby exerting immunomodulatory activities; through these mechanisms, macrophages play important roles in the body's specific immune responses. Moreover, macrophages can secrete cytotoxic molecules, functioning as natural killer cells. In a contrasting role, macrophages can secrete cytokines that regulate cell growth and differentiation [14]. Thus, macrophages are critical mediators of various immune functions within the body.

CBG is one of the primary pharmacologically active components of toad skin extracts and has been used for a long time as a traditional Chinese medicine [15]. In this study, we examined the functions of CBG in the immune system. Cellular immunity is an important immune response involving defense against pathogens and removal of infected cells. Two of the main functions of cellular immunity are phagocytosis of intracellular pathogens and release of large amounts of cytokines. Many of the changes in immunity involve alterations in activated T cells and cell proliferation. Therefore, T-cell proliferation capacity is an important indicator of the level of cell immunity [16]. In this study, our results showed that CBG enhanced lymphocyte proliferation. Moreover, combined treatment with ConA or LPS did not enhance these effects; these data suggested that the mechanisms through which CBG affects lymphocyte proliferation may be different from those of other drugs.

Lymphocytes achieve immune function via different subpopulations of cells. T cells can be divided into CD4^+^ T cells (Th cells) and CD8^+^ T cells (Tc cells). The dynamic balance between CD4^+^ T and CD8^+^ T cells is critical for maintaining the body's immune status [17]. Indeed, a reduction in the CD4^+^/CD8^+^ cell ratio may indicate the presence of infection. In the present study, we showed that CBG treatment significantly increased the percentages of CD4^+^/CD8^−^, CD4^−^/CD8^+^, and CD4^+^/CD8^+^ T cells.

Physiological or pathological apoptotic stimuli are correlated with cell cycle progression [18]. Unscheduled proliferation and escape from cell death signals may lead to malignant transformation [19]. The results of this study showed that CBG could increase the percentage of spleen lymphocytes in the S phase of the cell cycle, as measured by the increase in the DNA synthesis phase number. Thus, CBG promoted lymphocyte transformation from the G_0_/G_1_ phase to the S phase, providing the necessary conditions for the transition from S phase into G_2_/M phase.

Cytokines are proteins that are secreted by activated immune cells and transmit signals to regulate immune function; the level of cytokine secretion directly reflects the immune status. In our study, we found that CBG treatment enhanced the secretion of IL-2 and IL-10, suggesting enhancement of immune function. IL-2 has been shown to promote T-cell activation and proliferation, enhance cellular and humoral response, and serve as a tool for evaluating immune function [20]. In addition, IL-10 is a highly effective anti-inflammatory cytokine that functions by blocking proinflammatory cytokines and chemokines. Thus, our data further support the immune-enhancing functions of CBG through stimulation of cytokine secretion.

In this study, we also examined the effects of CBG on phagocytosis of macrophages. Macrophages are the first line of defense in preventing microorganisms from invading the body and in triggering anticancer immunity. After phagocytosis of pathogens, macrophages function in antigen presentation through expression of higher levels of costimulatory molecules, which modulate the interaction between T cells and macrophages. Our study showed that CBG treatment enhanced the phagocytosis function of macrophages, exhibiting increased consumption of* S. aureus*. Therefore, these data suggested that CBG has the ability to increase the innate immune response.

In conclusion, our results showed that CBG could enhance lymphocyte proliferation, increase the CD4^+^/CD8^+^ ratio, promote lymphocyte transformation from the G_0_/G_1_ to S phase, enhance the secretion of IL-2 and IL-10, and stimulate macrophage phagocytosis. Thus, these results demonstrated that CBG could enhance the immunomodulatory activity of immune cells in mice. This is the first time that the theoretical basis for the development and use of CBG has been provided. CBG nonspecific immunity and specific cellular immune responses in mice. The mechanism may be by raising the CD4^+^, CD8^+^ T lymphocyte subsets percentage. However, its mechanism still remains unclear and requires further research.

## Figures and Tables

**Table 1:** Effects of CBG on the expression of CD4 and CD8 in spleen lymphocytes.

CBG (*μ*g/mL)	CD4^+^/CD8^−^ (%)	CD4^−^/CD8^+^ (%)	CD4^+^/CD8^+^ (%)	CD4^+^/CD8^+^
0	0.085 ± 0.008	0.1.838 ± 0.141	0.857 ± 0.072	0.046 ± 0.004
0.0125	0.190 ± 0.014^*∗∗*^	3.500 ± 0.114^*∗∗*^	0.993 ± 0.073	0.054 ± 0.003^*∗∗*^
0.05	0.288 ± 0.025^*∗∗*^	4.825 ± 0.195^*∗∗*^	1.537 ± 0.169^*∗∗*^	0.059 ± 0.004^*∗∗*^
0.15	0.425 ± 0.019^*∗∗*^	5.075 ± 0.109^*∗∗*^	0.425 ± 0.019^*∗∗*^	0.084 ± 0.004^*∗∗*^
0.25	0.263 ± 0.020^*∗∗*^	5.520 ± 0.143^*∗∗*^	0.263 ± 0.020^*∗∗*^	0.048 ± 0.005

Statistical significance to corresponding control is marked with (*∗∗*) (*P* < 0.01).

**Table 2:** Effects of CBG on the cell cycle distribution in spleen lymphocytes.

CBG (*μ*g/mL)	G_1_ (%)	S (%)	G_2_ (%)
0	97.11 ± 0.78	0.91 ± 0.49	1.99 ± 0.07
0.0125	92.36 ± 0.73^*∗∗*^	6.06 ± 0.63^*∗∗*^	1.65 ± 0.22^*∗∗*^
0.05	92.36 ± 0.6^*∗∗*^	6.68 ± 0.54^*∗∗*^	0.96 ± 0.13^*∗∗*^
0.15	90.70 ± 0.84^*∗∗*^	7.24 ± 0.73^*∗∗*^	2.061 ± 0.29
0.25	90.83 ± 0.84^*∗∗*^	6.36 ± 0.29^*∗∗*^	2.81 ± 0.19^*∗∗*^

Statistical significance to corresponding control is marked with (*∗∗*) (*P* < 0.01).

## References

[1] Kuang H., Xia Y., Liang J., Yang B., Wang Q., Wang X. (2011). Structural characteristics of a hyperbranched acidic polysaccharide from the stems of *Ephedra sinica* and its effect on T-cell subsets and their cytokines in DTH mice. *Carbohydrate Polymers*.

[2] Zhao Y.-L., Wang J.-B., Shan L.-M., Jin C., Ma L., Xiao X.-H. (2008). Effect of radix isatidis polysaccharides on immunological function and expression of immune related cytokines in mice. *Chinese Journal of Integrative Medicine*.

[3] Mosmann T. R., Coffman R. L. (1989). TH1 and TH2 cells: different patterns of lymphokine secretion lead to different functional properties. *Annual Review of Immunology*.

[4] Leiro J. M., Castro R., Arranz J. A., Lamas J. (2007). Immunomodulating activities of acidic sulphated polysaccharides obtained from the seaweed *Ulva rigida* C. Agardh. *International Immunopharmacology*.

[5] Zhang Y., Feng J., Ye W., Tian H., Jiang R. (2014). Crystal structure and anticancer properties of cinobufagin 3-hemisuberate methyl ester. *Chinese Journal of Structural Chemistry*.

[6] Waiwut P., Inujima A., Inoue H., Saiki I., Sakurai H. (2012). Bufotalin sensitizes death receptor-induced apoptosis via Bid- and STAT1-dependent pathways. *International Journal of Oncology*.

[7] Zhai X., Lu J., Wang Y., Fang F., Li B., Gu W. (2014). Reversal effect of bufalin on multidrug resistance in K562/VCR vincristine-resistant leukemia cell line. *Journal of Traditional Chinese Medicine*.

[8] Lan Y. N. (2002). Long-term efficacy of cinobufacini for treatment of chronic hepatitis B. *Chinese Journal of Clinical Hepatology*.

[9] Zhang J., Sun Y., Liu J.-H., Yu B.-Y., Xu Q. (2007). Microbial transformation of three bufadienolides by *Nocardia* sp. and some insight for the cytotoxic structure-activity relationship (SAR). *Bioorganic and Medicinal Chemistry Letters*.

[10] Yeh J.-Y., Huang W. J., Kan S.-F., Wang P. S. (2003). Effects of bufalin and cinobufagin on the proliferation of androgen dependent and independent prostate cancer cells. *Prostate*.

[11] Su Y. H., Yin X. C., Xie J. M., Gao B., Ling C. Q. (2003). Inhibition effects of three kinds of bufotoxins on human SMMC-7721 and BEL-7402 hepatoma cells lines. *Dier Jun Yi Daxue Xuebao*.

[12] Mosmann T. R., Sad S. (1996). The expanding universe of T-cell subsets: Th1, Th2 and more. *Immunology Today*.

[13] Parker D. C. (1993). T cell-dependent B cell activation. *Annual Review of Immunology*.

[14] Lee K. Y., Jeon Y. J. (2005). Macrophage activation by polysaccharide isolated from *Astragalus membranaceus*. *International Immunopharmacology*.

[15] Wang D.-L., Oi F.-H., Xu H.-L. (2010). Apoptosis-inducing activity of compounds screened and characterized from cinobufacini by bioassay-guided isolation. *Molecular Medicine Reports*.

[16] Xu H.-S., Wu Y.-W., Xu S.-F., Sun H.-X., Chen F.-Y., Yao L. (2009). Antitumor and immunomodulatory activity of polysaccharides from the roots of *Actinidia eriantha*. *Journal of Ethnopharmacology*.

[17] Germain R. N. (2002). T-cell development and the CD4–CD8 lineage decision. *Nature Reviews Immunology*.

[18] Siegers C.-P., Steffen B., Röbke A., Pentz R. (1999). The effects of garlic preparations against human tumor cell proliferation. *Phytomedicine*.

[19] Frantz D. J., Hughes B. G., Nelson D. R., Murray B. K., Christensen M. J. (2000). Cell cycle arrest and differential gene expression in HT-29 cells exposed to an aqueous garlic extract. *Nutrition and Cancer*.

[20] Lillehoj H. S., Kaspers B., Jenkins M. C., Lillehoj E. P. (1992). Avian interferon and interleukin-2: a review by comparison with mammalian homologues. *Poultry Science Reviews*.

